# Regulation of NUB1 Activity through Non-Proteolytic Mdm2-Mediated Ubiquitination

**DOI:** 10.1371/journal.pone.0169988

**Published:** 2017-01-18

**Authors:** Thomas Bonacci, Stéphane Audebert, Luc Camoin, Emilie Baudelet, Juan-Lucio Iovanna, Philippe Soubeyran

**Affiliations:** Centre de Recherche en Cancérologie de Marseille (CRCM), INSERM U1068, CNRS UMR 7258, Aix-Marseille Université and Institut Paoli-Calmettes, Parc Scientifique et Technologique de Luminy, Marseille, France; Augusta University, UNITED STATES

## Abstract

NUB1 (Nedd8 ultimate buster 1) is an adaptor protein which negatively regulates the ubiquitin-like protein Nedd8 as well as neddylated proteins levels through proteasomal degradation. However, molecular mechanisms underlying this function are not completely understood. Here, we report that the oncogenic E3 ubiquitin ligase Mdm2 is a new NUB1 interacting protein which induces its ubiquitination. Interestingly, we found that Mdm2-mediated ubiquitination of NUB1 is not a proteolytic signal. Instead of promoting the conjugation of polyubiquitin chains and the subsequent proteasomal degradation of NUB1, Mdm2 rather induces its di-ubiquitination on lysine 159. Importantly, mutation of lysine 159 into arginine inhibits NUB1 activity by impairing its negative regulation of Nedd8 and of neddylated proteins. We conclude that Mdm2 acts as a positive regulator of NUB1 function, by modulating NUB1 ubiquitination on lysine 159.

## Introduction

Post-translational modifications (PTMs) by ubiquitin and ubiquitin-like proteins (Ubls) have emerged as major regulators of cellular functions [1,2]. Ubiquitination involves the successive action of an ubiquitin-activating enzyme (E1), a conjugating enzyme (E2), and an ubiquitin ligase (E3), leading to the covalent attachment of ubiquitin on an internal lysine residue of the modified protein. E3s modify substrate proteins with a single ubiquitin molecule (mono-ubiquitination), several single ubiquitin molecules (multi-ubiquitination) or by eight different types of poly-ubiquitin chains (poly-ubiquitination), obtained through the sequential attachment of ubiquitin molecules using either one of the seven lysine residues or their N-terminal methionine. These ubiquitination patterns dictate specific outcomes for the substrate proteins such as degradation, protein interactions, sub-cellular localization and/or activity. Importantly, ubiquitination can be reversed through the action of specific deubiquitinating enzymes (DUBs).

Ubls proteins, such as Nedd8 (neural precursor cell expressed developmentally down-regulated 8), share a very similar ternary structure and are conjugated to their substrates by specific sets of E1 / E2 / E3 enzymes. Neddylation primarily occurs on cullins thereby enabling the activity of cullin-RING ligases (CRLs), the largest subfamily of E3 ubiquitin ligases. Following Neddylation of the cullin subunit, CRLs regulates the turnover of several key regulators of main cellular functions such as the cell cycle or cell death, gene transcription or signaling pathways [3].

Similarly to ubiquitin, Nedd8 is removed through the action of Nedd8 isopeptidases. However, levels of neddylated proteins are also regulated by NUB1 (Nedd8 ultimate buster 1). NUB1 has been identified as a Nedd8-interacting protein, whose interaction promotes proteasomal degradation of Nedd8 and of neddylated proteins [4,5]. It is an interferon-inducible protein containing a ubiquitin-like (UBL) domain in its N-terminus, and two ubiquitin-associated (UBA) domains in its C-terminus. A longer isoform NUB1L possesses an extra UBA domain and facilitates proteasomal degradation of the Ubl FAT10 and FAT10-conjugated proteins [6]. NUB1L also interacts with the aryl hydrocarbon receptor interacting protein-like 1 (AIPL1), whose mutations lead to the inherited blindness Leber congenital amaurosis (LCA) and abolishes its interaction with NUB1L, suggesting that NUB1L could be involved in the pathogenesis of LCA [7]. NUB1 also suppresses the formation of Lewy body-like inclusions, by binding and targeting synphilin-1 for proteasomal degradation [8]. More recently NUB1 has been implicated in Huntington disease, a neurodegenerative disorder due to accumulation of a mutant huntingtin (mHTT) protein. This study showed that NUB1 reduces mHTT protein level by triggering its polyubiquitination and subsequent proteasomal degradation [9]. They also showed that interferon-β could be used as a treatment, by inducing NUB1 expression to enhance clearance of mHTT. Finally, NUB1 also promotes cytoplasm localization of the tumor suppressor p53, by reducing its Neddylation while conversely stimulating its ubiquitination, leading to the inhibition of p53 transcriptional activity [10]. Therefore, NUB1 is implicated in the regulation of substrate proteins implicated in pathologies including cancer and neurodegeneration. However, so far, no regulatory mechanisms of NUB1 functions has been described. In this study, we show that NUB1 is ubiquitinated by the oncogenic E3 ubiquitin ligase Mdm2, one of the main negative regulators of the p53 tumor suppressor [11,12]. Interestingly, we found that Mdm2-mediated ubiquitination of NUB1 is not a proteolytic signal. Our results rather suggest that Mdm2 specifically ubiquitinates NUB1 on lysine 159 and that this modification is required for NUB1 functions. Indeed, mutant NUB1 in which this lysine residue is mutated to arginine was no more able to negatively regulate Nedd8 and neddylated proteins.

## Materials and Methods

### Cell culture and reagents

HEK-293T (human embryonic kidney cells) were purchased from the American Type Culture Collection (Manassas, VA, USA) and grown according to ATCC recommendations. The following reagents were used: Ni^2+^-NTA agarose beads (Qiagen), Imidazole (Sigma-Aldrich), Cycloheximide (Sigma-Aldrich), MG132 (Santa Cruz).

### Plasmids and transfections

The full-lenght cDNA for human NUB1 was subcloned into a pcDNA3-Myc vector using XbaI restriction site. 6His-Flag-Ubiquitin and 6His-Flag-Nedd8 have been described in [13]. Mdm2 wild type and catalytically inactive mutant C462A expression plasmids were kind gifts of Dr Dimitris Xirodimas and have been described previously [14]. The 6His-Flag-Ubiquitin K0 mutant was generated by subcloning Ubiquitin K0 cDNA (kind gift of Dr Xirodimas) between BamHI and EcoRV digestion sites of the pCCL-WPS-PGK lentiviral vector. All cloning products were verified by automated sequencing. Cells were transiently transfected using Lipofectamine 2000 reagent (Invitrogen) according to the manufacturer's instructions.

### Site-directed mutagenesis

6HF-Ubiquitin and Myc-NUB1 mutants were generated by PCR using QuickChange Site-Directed Mutagenesis (Stratagene). The following forward oligonucleotides were used (mutated codons are underlined):

Ubiquitin K11R: 5’-AACCCTTACGGGGAGGACCATCACCCTCG-3’

Ubiquitin K48R: 5’-AGAGACTGATCTTTGCTGGCAGGCAGCTGGAAGA -3’

NUB1 K134R: 5’-GGACTTCAAGAAAATTATATCAAAATTGTCATAAATAAGAGGCAA CTACAACTAGGGAAA-3’

NUB1 K159R: 5’- AAAGCGATGGTGCTTGAACTAAGACAATCTGAAGAGGAC-3’

All constructs were verified by automated sequencing.

### Co-immunoprecipitation

HEK-293T cells were transfected in six-well plates and lysis was performed 24 h post-transfection in HEPES lysis buffer (50 mM HEPES, 150 mM NaCl, 1 mM EDTA, 10% glycerol, 1% Triton X-100, 25 mM NaF, 10 μM ZnCl_2_, plus protease inhibitors cocktail (Roche, 1:200), 10 mM N-ethylmaleimide (NEM) and 2 mM phenylmethylsulfonyl fuoride (PMSF)). Pre-cleared lysates were incubated with the appropriate antibodies for 2 h at 4°C. Immune complexes were precipitated with protein G-Sepharose beads and separated through SDS-PAGE.

### N-Ethylmaleimide sensitivity assay

Cells were transfected in six-well plates and solubilized in a hypotonic / Tween-20 buffer (50 mM NaH_2_PO_4_, 150 mM NaCl, 1% Tween-20, 5% Glycerol, pH 8.0) supplemented or not with 10 mM NEM. After 10 mn of incubation on ice, lysates were centrifuged for 10 mn at 4°C and at maximum speed, pellets were discarded and lysates incubated on ice for another 30 mn. Cell extracts were then prepared and analyzed by SDS-PAGE.

### Isolation of 6His-Ubiquitinated proteins

HEK-293T cells were transfected in 10 cm dishes and harvested in PBS 24 h post-transfection. 80% of cell suspension was lysed in 6 M Guanidine-HCl containing buffer to pull down 6His-Ubiquitinated proteins on Ni^2+^-NTA agarose beads (Qiagen), while the remaining 20% was used to prepare whole cell extracts. Ni^2+^ pull down eluates and cell extracts were separated through SDS-PAGE and analyzed by western blot.

### Cycloheximide treatment

Cells in 10 cm dishes were transfected as indicated, and seeded in 6 well plates at 50% confluence 24 h post-transfection. The day after, cells were incubated with 100 μg/ml of cycloheximide for the indicated time. Cell extracts were analyzed by SDS-PAGE.

### Identification of NUB1 ubiquitinated lysine(s) and mass spectrometry

Two sets of ten 10 cm dishes of HEK-293T were transfected with 2 μg of 6HF-Ubiquitin and 2 μg of Myc-NUB1 expression constructs, either alone or with 2 μg of Mdm2. At 48 h post-transfection, cells were harvested in PBS containing 10 mM NEM and centrifuged for 3 mn at 2000 rpm at 4°C. Cell pellets were lysed and solubilized with 25 ml of buffer 1 (6 M Guanidine-HCl, 0.1 M Na_2_HPO_4_ / NaH_2_PO_4_, 0.01 M Tris/HCl, pH 8.0 plus 10 mM imidazole and 10 mM β-mercaptoethanol (β-ME)). Lysates were sonicated three times for 30 sec with a 1 min break between pulses. Protein concentration was determined by Bradford assay (Bio-Rad), and Ni^2+^-NTA agarose beads (Qiagen) pre-washed with buffer 1 was added with a ratio of 2 μl of resin for 1 mg of proteins. Samples were rotated at room temperature for 4 h, and beads were successively washed for 5 min in each step at RT with 1 ml of each of the following buffers: buffer 1; buffer 2 (8 M urea, 0.1 M Na_2_HPO_4_ / NaH_2_PO_4_, 0.01 M Tris/HCl, pH 8.0, 10 mM β-ME); buffer 3 (8 M urea, 0.1 M Na_2_HPO_4_ / NaH_2_PO_4_, 0.01 M Tris/HCl, pH 6.3, 10 mM β-ME) plus 0.2% Triton X-100; buffer 3 and then buffer 3 plus 0.1% Triton X-100. Beads were washed two times more with 1 ml of prechilled buffer 2 (50 mM NaH_2_PO_4_, 150 mM NaCl, 1% Tween20, 5% Glycerol, pH 8.0) plus 10 mM imidazole. 6HF-Ubiquitinated proteins were eluted for 2 h at 4°C in 600 μl of buffer 2 plus 250 mM imidazole. Eluates were then incubated with 15 μg of anti-Myc 9E10 antibody and rotated at 4°C overnight. The day after, 30 μl of protein G-sepharose beads were added and samples were rotated for 1 h at 4°C. Beads were washed twice with 500 μl of prechilled buffer 2. 6HF-Ubiquitinated Myc-NUB1 was eluted by rotating the beads at 4°C for 2 h 30 min in 100 μl of buffer 2 containing 1 μg/μl of Myc peptide. Eluates were collected and analyzed by mass spectrometry (S1 File).

### Huntingtin degradation assay

293T cells in 10 cm dishes were transfected with GFP tagged mutant Huntingtin protein, HTT97, in combination with NUB1 WT or K159R mutant. 24h after transfection, cells were harvested and seeded into six-well plates. 24h later, cells were treated with 100μM Cycloheximide for different laps of time from 0 to 36h. Cells were solubilized in lysis buffer and centrifugation cleared lysates were subjected to SDS-PAGE followed by western blot analysis of GFP-HTT97 NUB1 and actin expression. Densitometry analysis from three independent experiments has been performed using Image J software and the data used for calculation (S2 File).

### Western blot and antibodies

Proteins were resolved by SDS-PAGE, transferred to nitrocellulose filters, revealed with ECL and detected using a Fusion FX7 imager (Vilber-Lourmat, France). SNAP i.d. protein detection system (Millipore) was used for Flag (ubiquitin) and β-Tubuline immunoblots. The following antibodies were used: mouse monoclonal anti-Myc 9E10 (produced in the laboratory), mouse monoclonal anti-Mdm2 (MABE331, Millipore), mouse monoclonal anti-β-Tubulin (T4026, Sigma), rabbit polyclonal anti-NUB1 (BML-PW9685, Enzo), mouse monoclonal anti-Flag (M2, Sigma-Aldrich).

## Results

### Mdm2 interacts with NUB1 and triggers its ubiquitination

**Fig 1:**
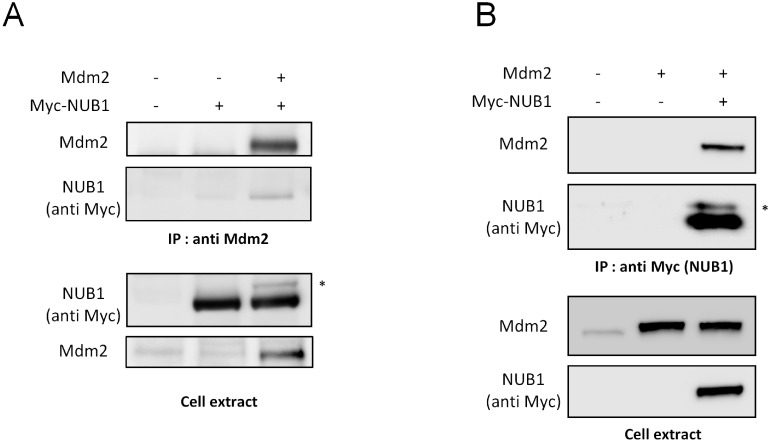
Mdm2 interacts with NUB1. (A) Lysates from HEK-293T cells expressing Myc-NUB1 alone or together with Mdm2 were subjected to immunoprecipitation with an anti-Mdm2 antibody. Proteins were separated through SDS-PAGE and NUB1 was detected by western blotting using an anti-Myc antibody (9E10). Amount of precipitated Mdm2 and expression levels of both proteins in cell extracts were controlled. (B) Lysates from HEK-293T cells expressing Mdm2 alone or together with Myc-NUB1 were subjected to immunoprecipitation with the anti-Myc 9E10 antibody. Immunoprecipitates and cell extracts were analyzed by western blotting using an anti-Mdm2 and anti-Myc antibodies. * Shifted NUB1.

**Fig 2:**
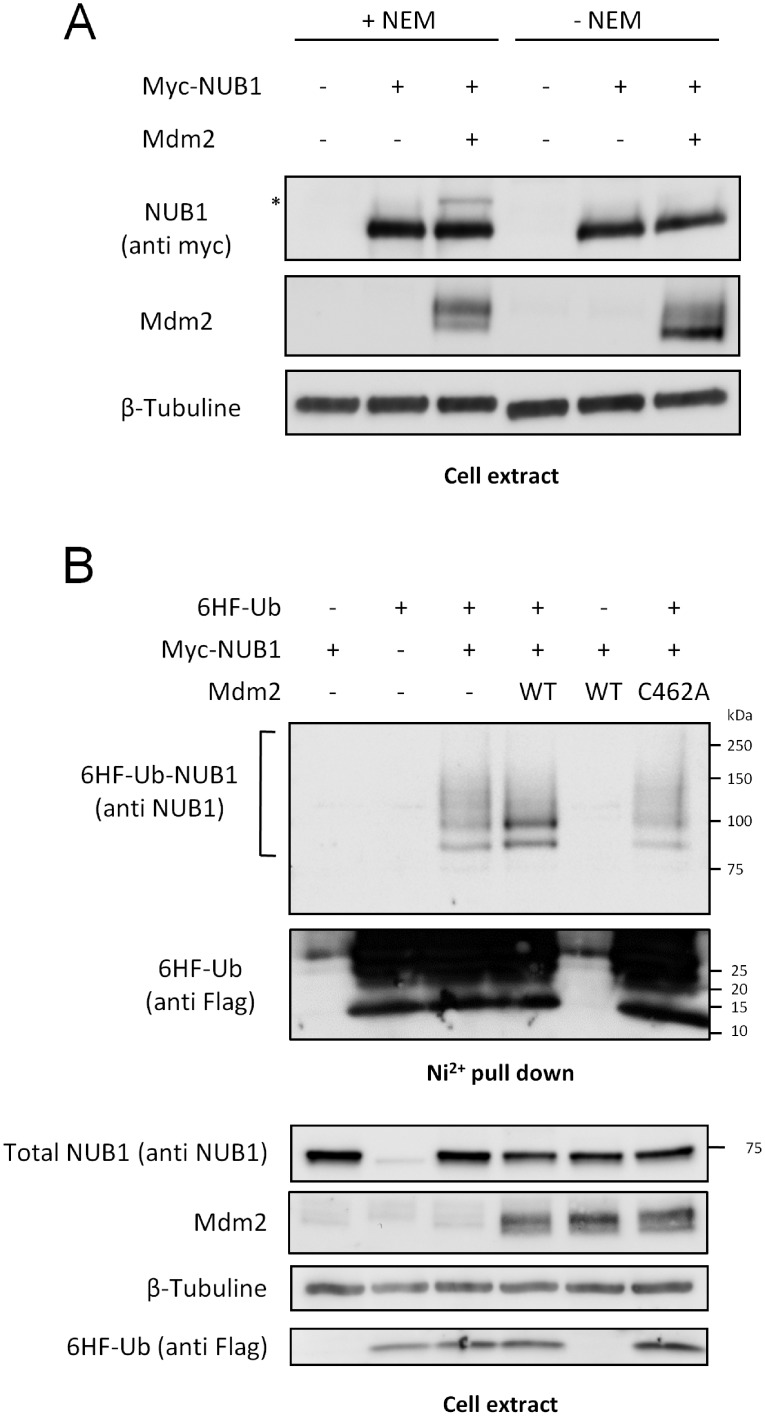
Mdm2 triggers the ubiquitination of NUB1. (A) HEK-293T cells were transfected with Myc-NUB1 alone or together with Mdm2, and twenty-four hours later lysates were prepared using an hypotonic / Triton X-100 buffer supplemented or not with N-Ethylmaleimide (NEM). Proteins were separated through SDS-PAGE and NUB1 was detected by western blotting using the anti-Myc antibody. (B) HEK-293T cells were transfected with Myc-NUB1 alone or together with wild type (WT) or catalytically inactive (C462A) Mdm2. Lysates were prepared and proteins were separated through SDS-PAGE and analyzed by western blotting with the appropriate antibodies.

Considering that NUB1 belongs to the p53 network, and because of its role regarding neddylated proteins, we wondered if it could also be targeted by one of the major p53 ubiquitin ligase, Mdm2. Therefore, we have studied the potential interaction between these two proteins. To this end, we have performed reciprocal co-immunoprecipitation experiments of over expressed proteins in transfected HEK-293T cells. As shown in Fig 1, over expressed NUB1 co-precipitated with over expressed Mdm2 (Fig 1A) and Mdm2 co-precipitated with NUB1 (Fig 1B), suggesting that NUB1 is a new potential Mdm2-interacting protein. Moreover, we have observed that Mdm2 co-expression led to the appearance of a heavier form of NUB1 (Fig 1, asterisks), which could correspond to NUB1 ubiquitination. To test this hypothesis, cell extracts from HEK-293T expressing NUB1 and Mdm2 were prepared using a lysis buffer supplemented or not with N-Ethylmaleimide (NEM), an inhibitor of deubiquitinating enzymes (DUBs). As shown in Fig 2A, the heavier form of NUB1 was lost when cell extracts were prepared without NEM. This suggests that the shifted NUB1 was indeed a substrate for DUBs activity *in cellulo* and that it corresponded to ubiquitinated NUB1. To further characterize Mdm2-mediated ubiquitination of NUB1, we have used an *in cellulo* ubiquitination assay. HEK-293T cells were transfected with Myc-NUB1, alone or together with Mdm2, along with a two-tagged (hexahistidine and Flag) version of ubiquitin (6HF-Ub). 6HF-Ubiquitinated proteins were isolated using Ni^2+-^NTA Agarose resin under fully denaturing conditions. Western blot analysis using an anti-NUB1 antibody detected a ladder of ubiquitinated forms of NUB1 with molecular weights higher than 75 kDa. Mdm2 over expression stimulated NUB1 ubiquitination between 75 and 100 kDa, corresponding probably to the conjugation of one and two ubiquitin moieties. Importantly, expression of a mutant ligase deficient form of Mdm2 (C462A) prevented the increased NUB1 ubiquitination observed with WT Mdm2 (Fig 2B, Ni^2+^ pull down panel). Sample of the cell extracts used for Ni2+ purification was used to reveal the non-modified NUB1, just below the 75 kDa molecular weight marker (Fig 2B, cell extract panel). All together, these data demonstrate that Mdm2 interacts with NUB1 to promote its ubiquitination.

#### Molecular characterization of Mdm2-mediated ubiquitination of NUB1

**Fig 3:**
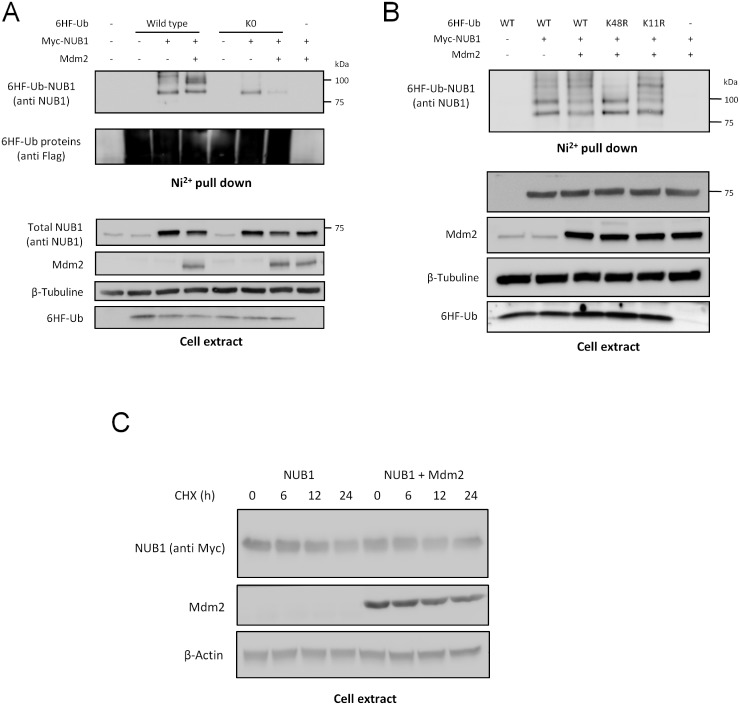
Molecular characterization of Mdm2-dependant ubiquitination of NUB1. (A) HEK-293T cells were transfected as indicated with Myc-NUB1, Mdm2 together with wild type (WT) or lysine-less mutant (K0) 6HF-Ubiquitin expressing constructs. Twenty-four hours post-transfection, 80% of cells were lysed in Guanidine-HCl containing buffer and 6HF-Ubiquitinated conjugates were isolated on Ni2+-NTA agarose beads. The remaining 20% were lysed in non-denaturing buffer to detect non-modified NUB1. Purified 6HF-Ubiquitinated proteins and cell extracts were resolved by SDS-PAGE and NUB1 was revealed by western blotting using an anti-NUB1 antibody. (B) HEK-293T cells were transfected with Myc-NUB1 and Mdm2, together with wild type (WT), K48R or K11R mutants 6HF-Ubiquitin. Ubiquitination of NUB1 was analyzed as in (A). (C) HEK-293T cells were transfected in 10-cm dishes with Myc-NUB1 alone or together with Mdm2. Twenty-four hours post-transfection, cells were split into six-well plates and allowed to rest overnight. Cells were then treated by supplementation with 100 μg/ml of cycloheximide (CHX) in their culture medium for the indicated time lapses. NUB1 stability was monitored by western blotting using the anti-Myc 9E10 antibody.

In order to discriminate between NUB1 poly- or multi-ubiquitination, we have studied the effect of Mdm2 on NUB1 ubiquitination using either WT ubiquitin or a K0 mutant ubiquitin in which all lysine residues are mutated into arginine residues to prevent the formation of polyubiquitin chains. Hence, although this K0 ubiquitin can still from linear polyubiquitin chains, the modification profiles exhibited by this K0 mutant ubiquitin are mainly mono- and multi-ubiquitination. As expected, the polyubiquitin-like profile of NUB1 obtained with WT ubiquitin expression was lost with K0 ubiquitin which displayed only the monoubiquitinated form of NUB1 (Fig 3A). Mdm2 is known to catalyze the ligation of K48-linked and K11-linked polyubiquitin chains to p53 [15]. To determine which kind of polyubiquitin chain Mdm2 uses to polyubiquitinate NUB1, we have used K48R and K11R mutants of ubiquitin. Whereas K48R mutant impaired the polyubiquitination of NUB1, K11R mutant did not (Fig 3B), indicating that NUB1 is conjugated to K48 type polyubiquitin chains. However, none of these mutant ubiquitins had a negative effect regarding the Mdm2-mediated NUB1 mono- and di-ubiquitination. These results strongly suggest that Mdm2 triggers the mono and di-ubiquitination of NUB1 whereas another ligase is in charge of its K48 polyubiquitination. Consistent with this, we could not observe any alteration of NUB1 stability upon over expression of Mdm2 (Fig 3C). We have followed the level of NUB1 after treating cells with the protein translation inhibitor Cycloheximide and we could not observe any significant difference when Mdm2 was over expressed (Fig 3C).

#### Mdm2 ubiquitinates NUB1 on lysine 159

**Fig 4:**
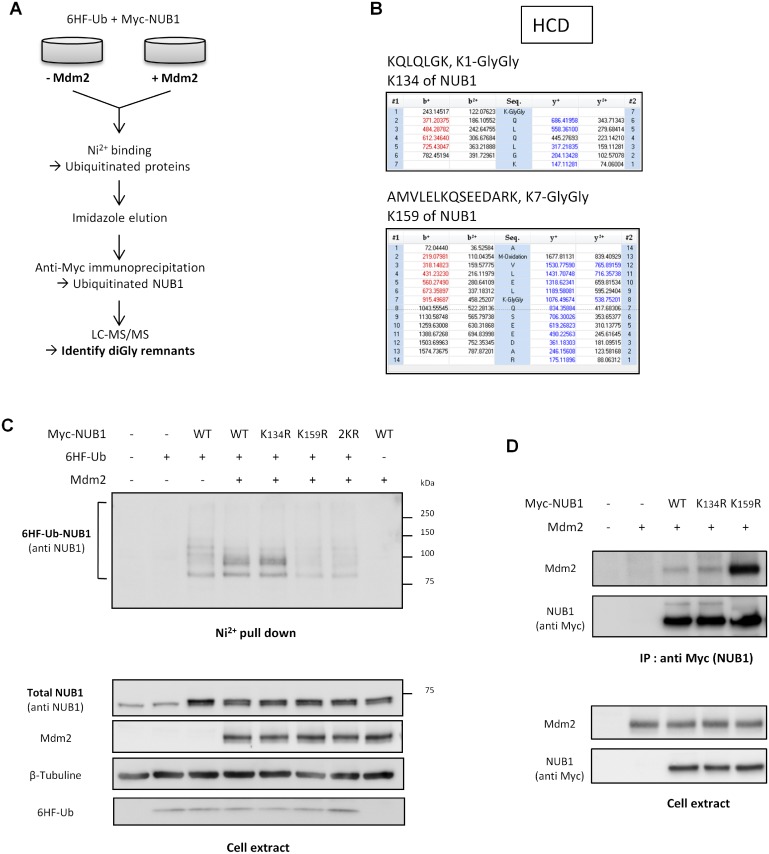
NUB1 is ubiquitinated by Mdm2 on lysine 159. (A) Workflow of the protocol used to identify NUB1 ubiquitinated lysine. (B) METTRE SPECTRES (C) HEK-293T cells were transfected with wild type (WT), K134R, K159R or K134,159R (2KR) mutants Myc-NUB1 together with 6HF-Ubiquitin and Mdm2 constructs. 6HF-Ubiquitinated proteins were isolated by Ni2+ pull down and NUB1 was revealed by western blotting using an anti-NUB1 antibody. (D) Lysates from HEK-293T cells expressing Mdm2 alone or together with wild type (WT), K134R or K159R mutants Myc-NUB1 were subjected to immunoprecipitation with the anti-Myc 9E10 antibody. Immunoprecipitates and input samples were analyzed by western blotting using an anti-Mdm2 antibody.

To gain insight into the function of NUB1 ubiquitination by Mdm2, we have intended to identify the lysine residue(s) of NUB1 ubiquitinated by Mdm2. To this end, we have designed a tandem affinity strategy to purify exclusively the ubiquitinated forms of NUB1 from transfected cells (Fig 4A). Two sets of HEK-293T were transfected with a mix of 6HF-Ubiquitin and Myc-NUB1 expressing constructs, with Mdm2 or an empty vector. Cells were first lysed under denaturing conditions, and lysates were incubated with Ni^2+^-NTA resin to pull down 6HF-Ubiquitinated proteins. 6HF-Ubiquitinated proteins were eluted in a non-denaturing buffer containing imidazole, and eluates were used to perform an anti-Myc immunoprecipitation to specifically isolate ubiquitinated NUB1 (see Materials and methods for details). Following trypsin digestion, peptides were analyzed by mass spectrometry to identify lysine residues containing the remnant di-glycine signature of ubiquitin. Lysine residues 134 and 159 of NUB1 were identified as potential ubiquitination sites (Fig 4B and S1 File). These residues were mutated into arginine, either individually (K134R and K159R) or simultaneously (2KR). Ubiquitination of these NUB1 mutants has been analyzed by Ni^2+^ pull down in HEK-293T cells (Fig 4C). Whereas K134R mutant NUB1 displayed an identical ubiquitination pattern to WT NUB1, K159R mutant showed a strong reduction of its ubiquitination with a disappearance of the di-ubiquitinated form of NUB1 normally induced by Mdm2 (Fig 4C). The same result was obtained with the 2KR mutant of NUB1. Importantly, lysine 159 of NUB1 is part of a coiled-coil domain [4], involved in protein to protein interactions. Thus we wondered if this mutation could impair NUB1 interaction with Mdm2 thereby explaining the loss of ubiquitination. The interaction between Mdm2 and single lysine mutants of NUB1 has been monitored by co-immunoprecipitation and, surprisingly, the K159R mutation strongly increased the NUB1-Mdm2 interaction, whereas K134R mutant NUB1 interacted with Mdm2 as efficiently as WT NUB1 (Fig 4D). Hence, the lack of Mdm2 mediated ubiquitination of NUB1 K159R could not be explain by loss of interaction between the two proteins, and it seemed that ubiquitination of NUB1 is necessary to destabilize the NUB1-Mdm2 complex.

#### Mdm2-mediated ubiquitination controls NUB1 functions towards neddylated proteins

**Fig 5:**
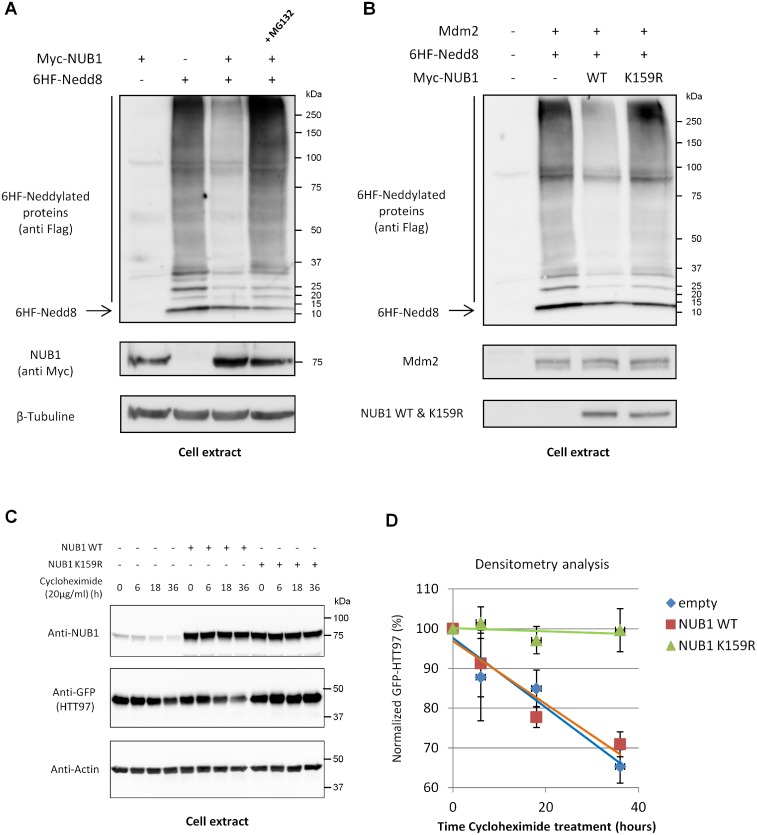
Mdm2-mediated ubiquitination controls NUB1 activity and is not a proteolytic signal. (A) HEK-293T cells were transfected with Myc-NUB1 and 6HF-Nedd8, alone or together. Twenty-four hours post-transfection, cells were treated with 30 μM of MG132 for 4 hours where indicated or with DMSO (vehicle) as a control. 6HF-neddylated proteins were revealed by western blotting using the anti-Flag M2 antibody. (B) HEK-293T cells were transfected with 6HF-Nedd8 and Mdm2, alone or together with WT or K159R Myc-NUB1. Twenty-four hours post-transfection, proteins were separated through SDS-PAGE and 6HF-neddylated proteins were revealed by western blotting using the anti-Flag M2 antibody. (C) HEK-293T cells were transfected with GFP-HHT97 in combination with Myc-NUB1 WT or K159R. 24 hours after transfection cells were dispatched in 6-wells plates and allowed to adhere for 12 more hours. Cells were then treated with 20 μg/ml of Cycloheximide and harvested at indicated time points. Amount of remaining HTT97 was evaluated by western blot of cell lysates (left panel). Densitometry analysis of three independent experiments have been performed and used to establish the half-life of HTT97 and to evaluate the impact of NUB1 WT or K159R expression (right panel).

Over-expression of Nedd8 in HEK-293T cells led to the appearance of a ladder of neddylated proteins, covering the entire range of molecular weights (Fig 5A). This patern likely represent atypical neddylation induced by Nedd8 overexpression, as described previously [16]. Consistent with previous studies, co-expression of NUB1 strongly decreased the abundance of these neddylated proteins [17]. However,this negative regulation of Nedd8 and of neddylated proteins was reversed by addition of the proteasome inhibitor MG132 (Fig 5A). It has been shown that proteome inhibition provokes the depletion of free ubiquitin in the cell also leading to atypical neddylation [18]. Thus, NUB1 regulates the amount of neddylated proteins in cells but this function is limited and may ma ybe exceeded. Next, we have analyzed the impact of Mdm2-mediated ubiquitination of NUB1 towards its Nedd8 regulation activity. To this end, HEK-293T cells were transfected with Nedd8 and Mdm2, along with WT or K159R mutant NUB1. As observed previously, co-expression of WT NUB1 led to a dramatic decrease of neddylated proteins (Fig 5B). However, this effect was completely lost with the K159R mutation. Hence, it seems that the Mdm2-mediated ubiquitination of NUB1 at lysine residue 159 is essential for the negative regulation of neddylation by NUB1. Besides its function regarding Nedd8, it has been shown that NUB1 has also the ability to accelerate the clearance of Huntingtin protein [9]. Therefore, we have studied if this specific function of NUB1 was also dependent on its ubiquitination at lysine 159. As shown in Fig 5C, whereas expression of NUB1 WT decreased the half-life of mutant Huntingtin protein, HTT97, NUB1 K159R mutant could not and, in contrary, it acted as a dominant negative as it could increase the stability of HTT97. Hence, lysine residue 159 of NUB1 is also important regarding its function as a negative regulator of mutant huntinting protein.

## Discussion

Because they are involved in roughly all cellular functions PTMs by ubiquitin and ubiquitin-like proteins play essential roles for the normal biology of the cell [1]. Consequently, failures within this system can promote or at least participate to a large variety of human pathologies, including cancers [19]. Ubiquitin has the most important role due to the extent of its substrates (potentially all cytoplasmic proteins) and their associated functions, and even a slight reduction of its cellular level has severe consequences [20]. Some ubls are also essential for life. Nedd8 is one of those essential ubls as its main function is to regulate the activity of the biggest family of ubiquitin ligases, the cullin ring ligases (CRLs) [3,21]. The number of substrates for these ligases is so tremendous that it is easily understandable why it is crucial for the cell to fine tune the level of Nedd8, and especially of neddylation, in order to avoid abnormal ubiquitination of many CRLs substrates.

NUB1 is an efficient and strong negative regulator of Nedd8 and of neddylated proteins [4,5] thereby regulating CRLs activity and the ubiquitination of their many substrates. Nedd8 can be conjugated to other substrates than cullins, thereby regulating their activity that may be also important, such as P53 which function is inhibited upon neddylation [14]. Whereas NUB1 is well-known to regulate Nedd8, regulation of NUB1 functions remained elusive until now. Because NUB1 acts as a platform interacting with Nedd8, neddylated proteins, and other NUB1 interacting proteins, its first level of regulation is at its expression level. Whereas it is expressed in all tissues, NUB1 was shown to be induced by inflammatory cytokines, such as interferon [4]. Here we describe another mechanism of regulation of NUB1 functions, dependent on a specific ubiquitination, which is not involved in proteasomal degradation.

Indeed, we have shown that Mdm2 triggers NUB1 ubiquitination independently of lysine 11 or 48 of ubiquitin (Fig 3B), two well know proteolytic polyubiquitin chains, leading us to hypothesize that ubiquitin conjugation to NUB1 could be a non-proteolytic signal. As a confirmation, we could never notice any difference in protein stability between WT et K159R NUB1 (Figs 4 and 5).

The exact mechanism remains uncertain but it is most likely that once NUB1 is mono or di-ubiquitinated at lysine 159, its UBA domains could interact with these ubiquitin moieties [22], thereby creating an intra or inter-molecular interaction of NUB1 with itself. These new interactions may impose a conformational change to NUB1 which would expose its Nedd8 interaction motifs. Hence, NUB1 would remain as an inactivated form as long as it is not ubiquitinated on lysine 159 residue.

Whereas we have been able to detect a potential ubiquitination of lysine 134 by mass-spectrometry, it did not seem to take part in the Mdm2 mediated ubiquitination of NUB1. Indeed, changing lysine residue 134 to arginine did not change the ubiquitination profile of NUB1 by Mdm2. Hence another ligase must be involved in the ubiquitination of this lysine. In addition, even though we have shown that Mdm2 mediates this particular ubiquitination of NUB1 on K159, it is not excluded that other ligase(s) could do it as well.

Importantly, we could also observe that ubiquitination of NUB1 at K159 is not only necessary for its neddylation regulatory function but also for the regulation of other proteins, independently of their neddylation, as mHTT protein has never been shown to be neddylated.

In conclusion, we have identified a new positive regulatory mechanism of NUB1 which involves its ubiquitination by Mdm2 at lysine residue 159. Interfering with this modification seriously impaired the main know function of NUB1, that is the negative regulation of Nedd8 pathway.

## Supporting Information

S1 File Mass spectrometry analysis of NUB1 to identify Mdm2 mediated ubiquitination sites on NUB1. (PPTX)

S2 File Impact of NUB1 WT and K159R on HTT97 half life. Excel data file for the calculation of HTT97 half life from three independent experiments.
(XLSX)
