# Supplementary material for: Regulation of NUB1 Activity through Non-Proteolytic Mdm2-Mediated Ubiquitination
Source: PLoS One. 2017 Jan 18;12(1):e0169988. doi: 10.1371/journal.pone.0169988 (PMC5242482; doi:10.1371/journal.pone.0169988)
Supplement: S1 File — (PPTX) [file pone.0169988.s001.pptx]

## Slide 1
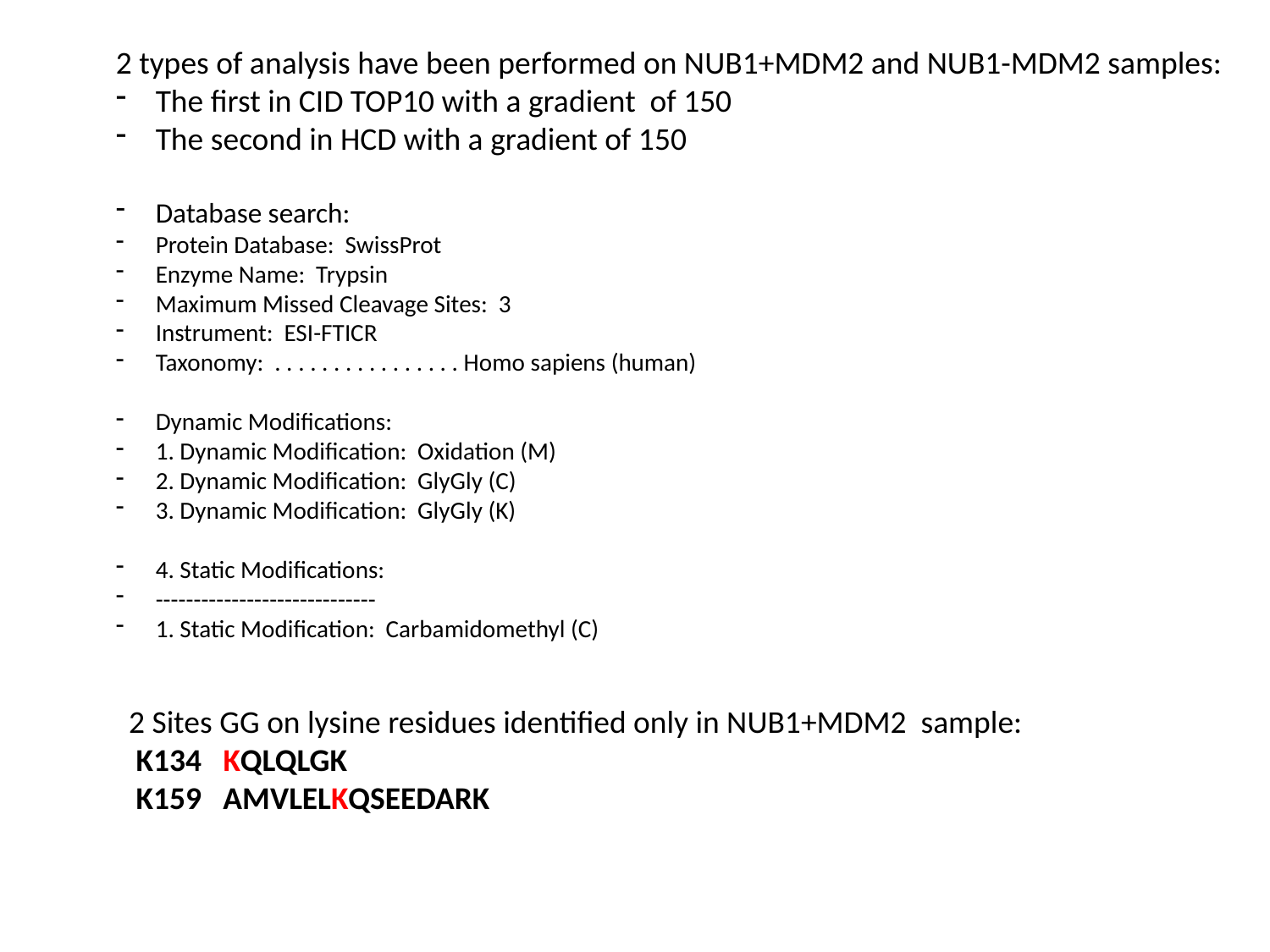

2 types of analysis have been performed on NUB1+MDM2 and NUB1-MDM2 samples:
The first in CID TOP10 with a gradient of 150
The second in HCD with a gradient of 150
Database search:
Protein Database: SwissProt
Enzyme Name: Trypsin
Maximum Missed Cleavage Sites: 3
Instrument: ESI-FTICR
Taxonomy: . . . . . . . . . . . . . . . . Homo sapiens (human)
Dynamic Modifications:
1. Dynamic Modification: Oxidation (M)
2. Dynamic Modification: GlyGly (C)
3. Dynamic Modification: GlyGly (K)
4. Static Modifications:
-----------------------------
1. Static Modification: Carbamidomethyl (C)
2 Sites GG on lysine residues identified only in NUB1+MDM2 sample:
 K134 KQLQLGK
 K159 AMVLELKQSEEDARK

## Slide 2
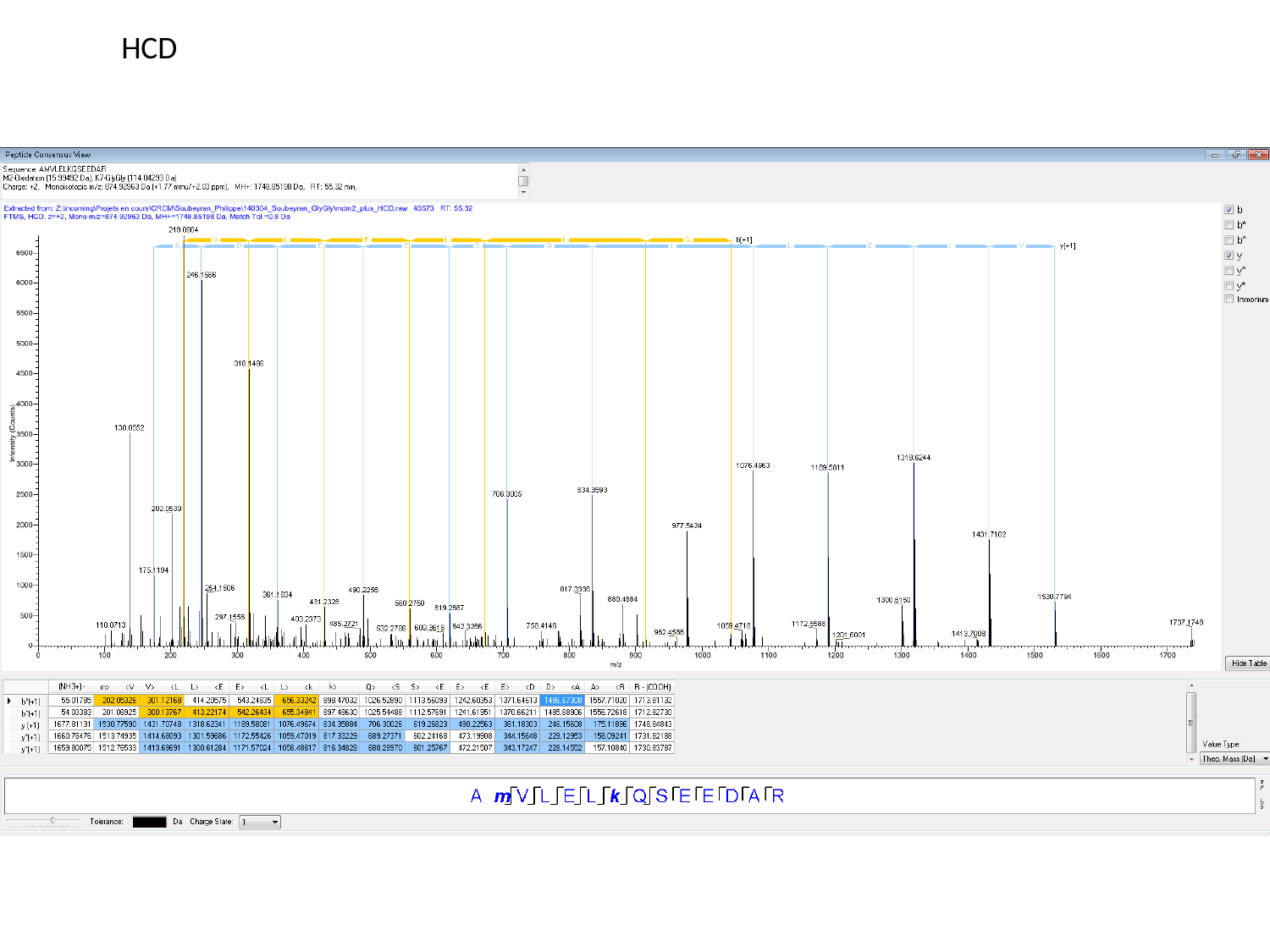

HCD

## Slide 3
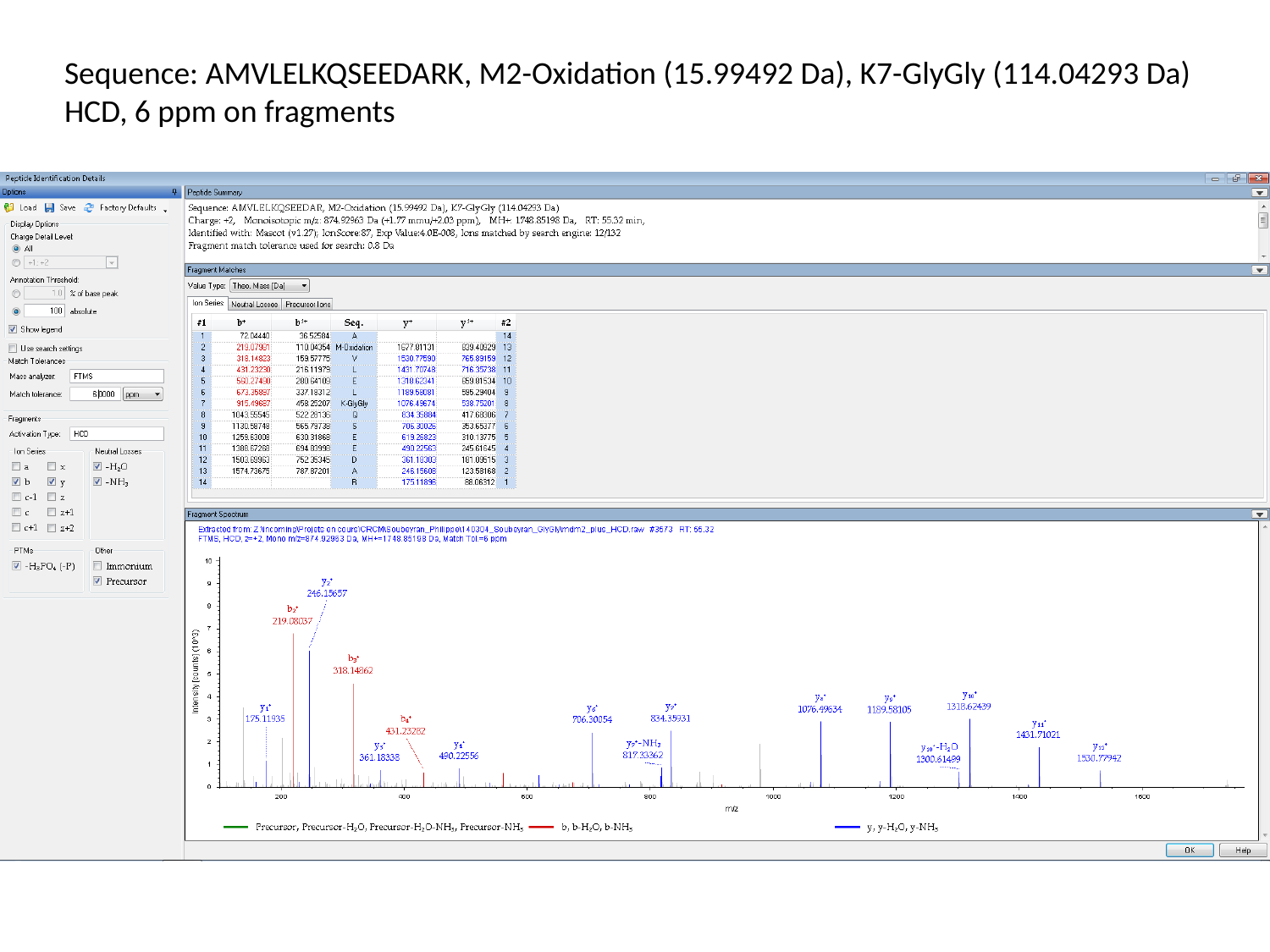

Sequence: AMVLELKQSEEDARK, M2-Oxidation (15.99492 Da), K7-GlyGly (114.04293 Da)
HCD, 6 ppm on fragments

## Slide 4
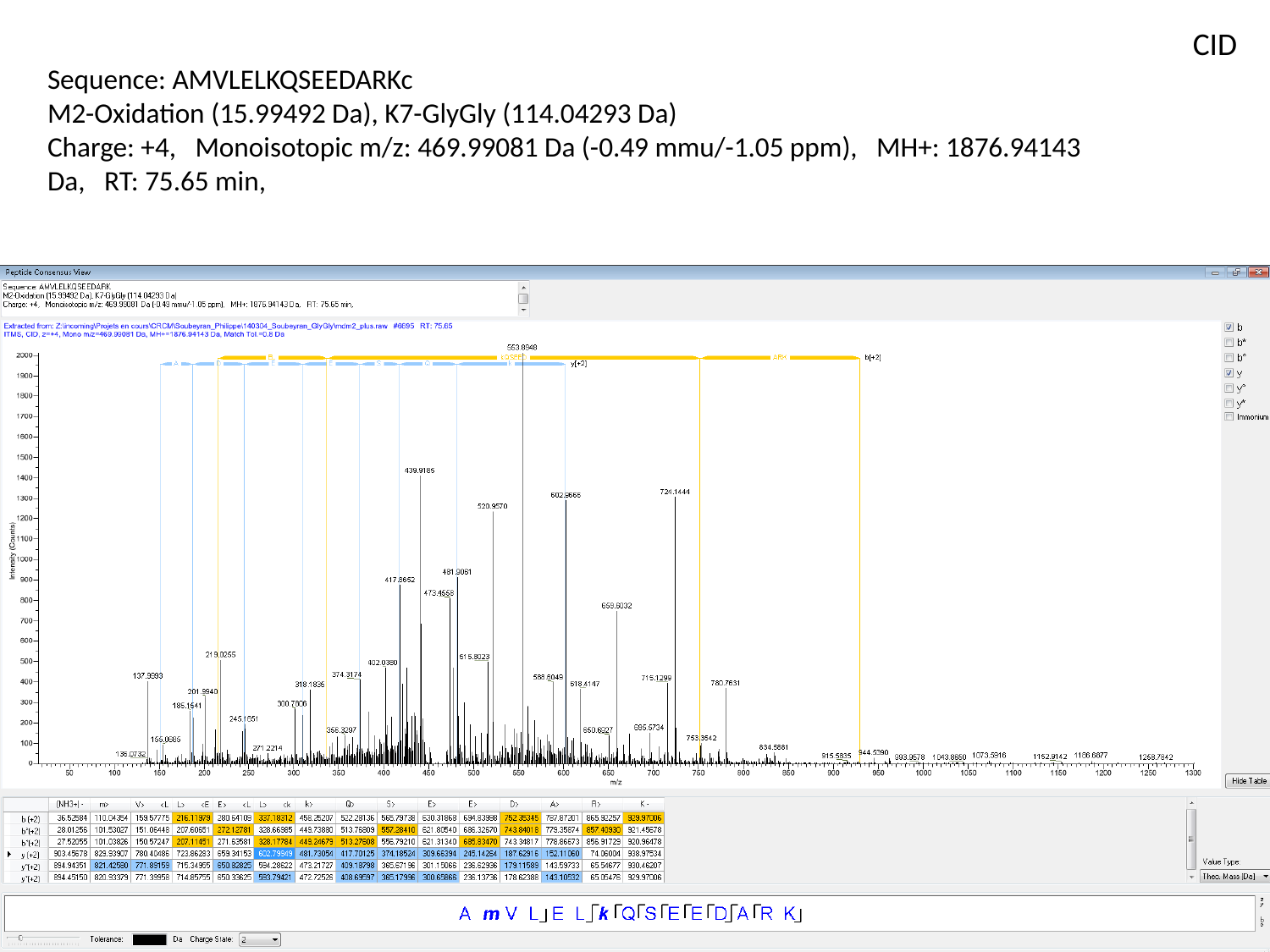

CID
Sequence: AMVLELKQSEEDARKc
M2-Oxidation (15.99492 Da), K7-GlyGly (114.04293 Da)
Charge: +4, Monoisotopic m/z: 469.99081 Da (-0.49 mmu/-1.05 ppm), MH+: 1876.94143 Da, RT: 75.65 min,

## Slide 5
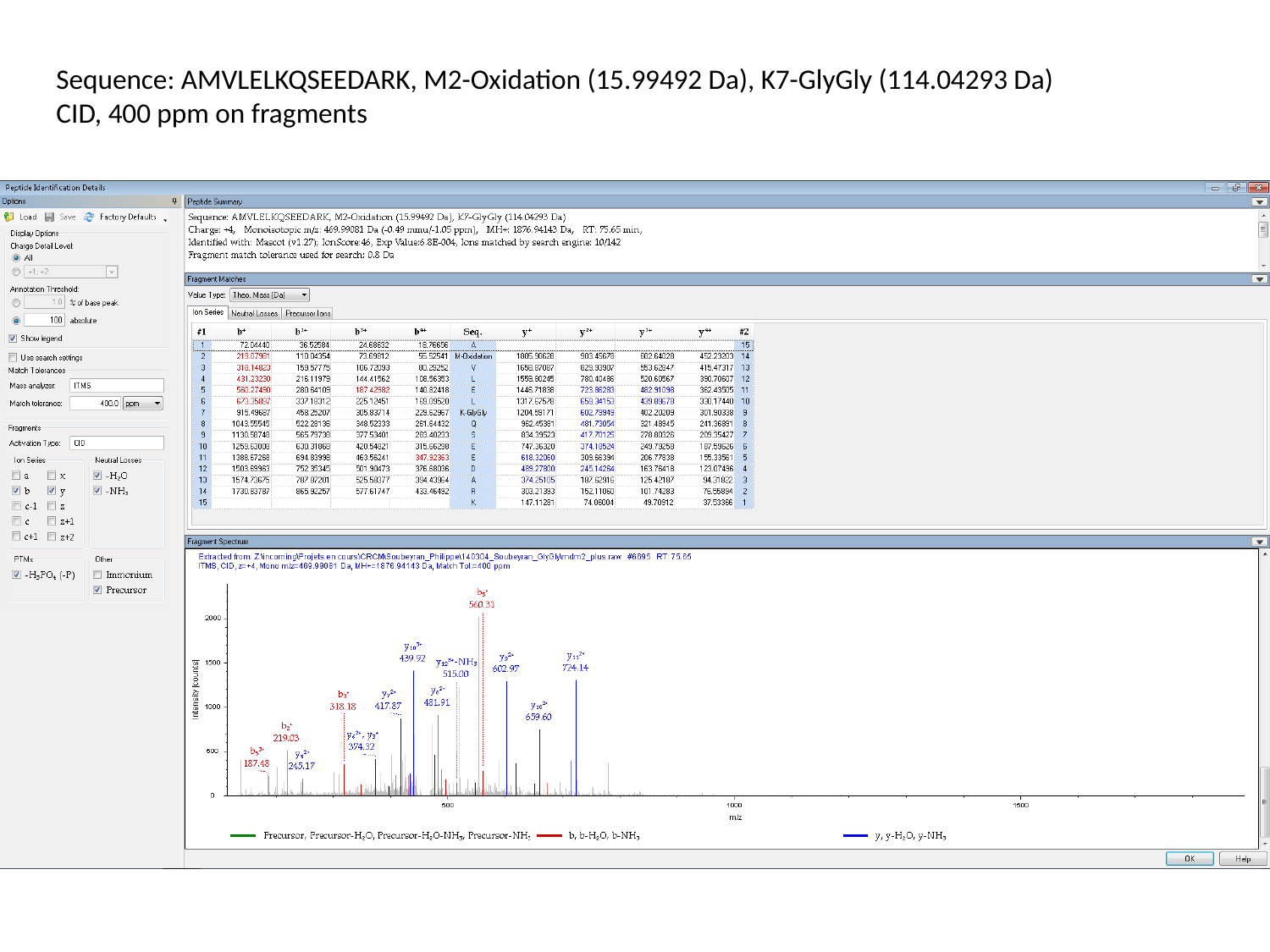

Sequence: AMVLELKQSEEDARK, M2-Oxidation (15.99492 Da), K7-GlyGly (114.04293 Da)
CID, 400 ppm on fragments

## Slide 6
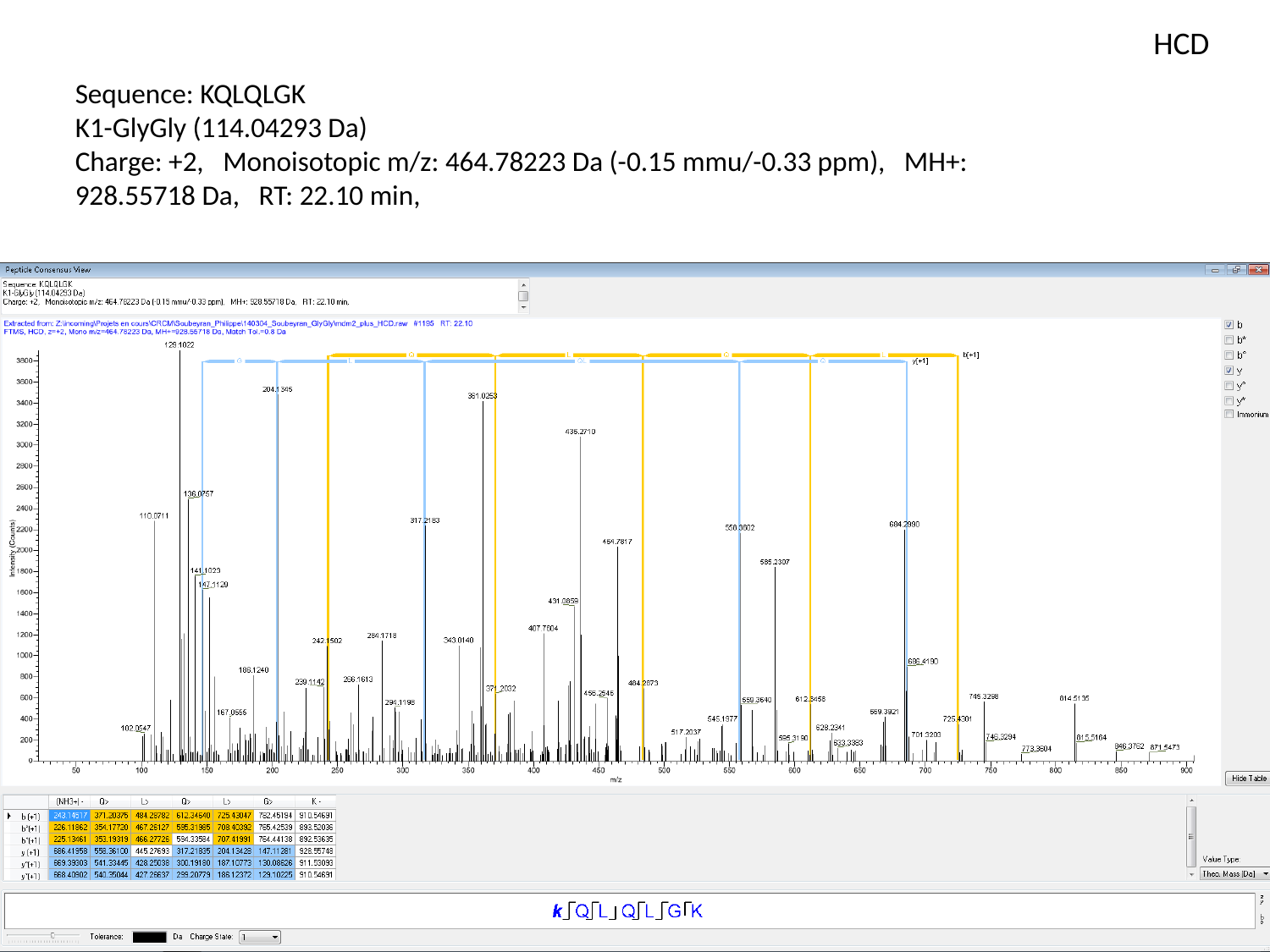

HCD
Sequence: KQLQLGK
K1-GlyGly (114.04293 Da)
Charge: +2, Monoisotopic m/z: 464.78223 Da (-0.15 mmu/-0.33 ppm), MH+: 928.55718 Da, RT: 22.10 min,

## Slide 7
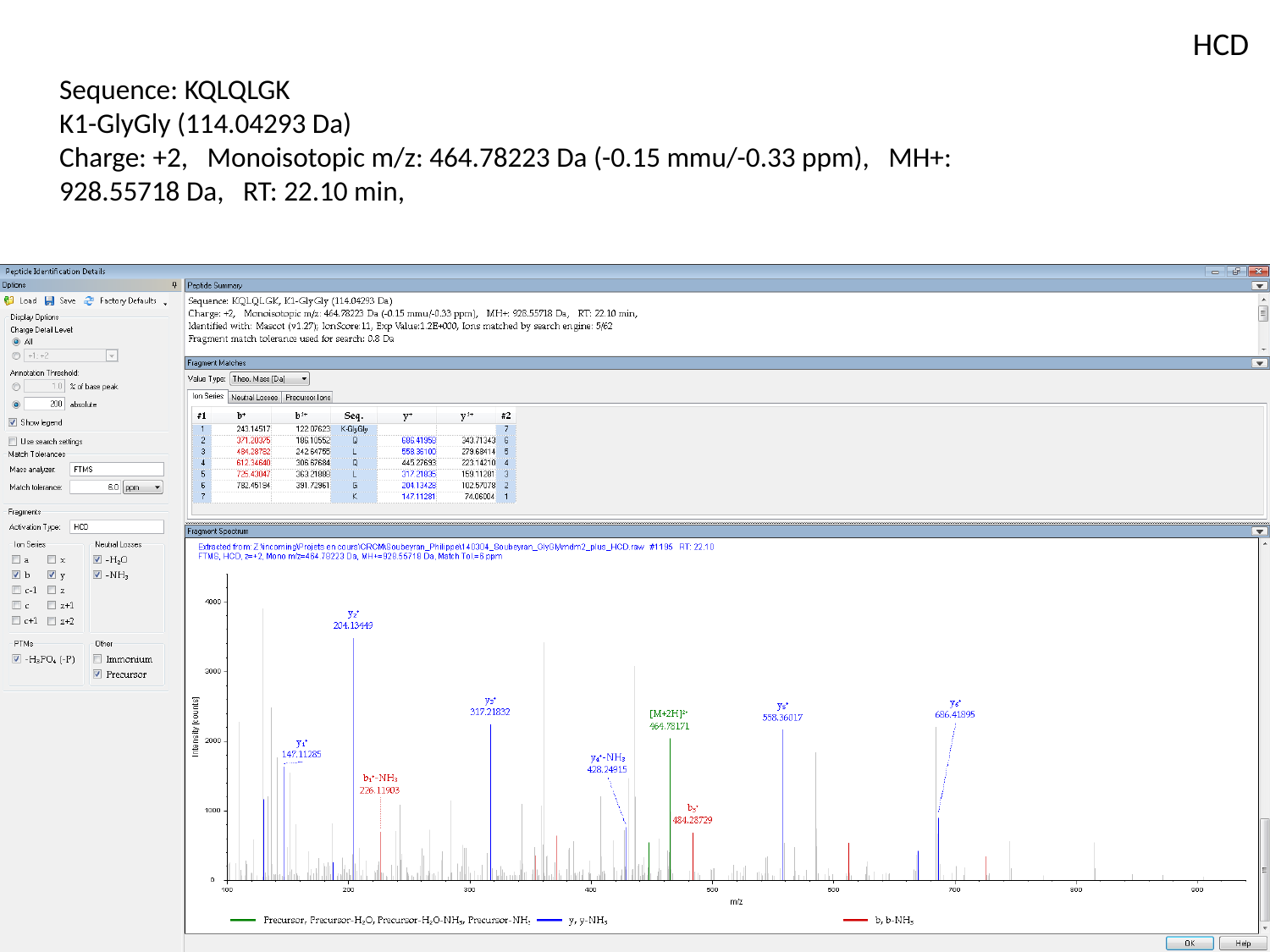

HCD
Sequence: KQLQLGK
K1-GlyGly (114.04293 Da)
Charge: +2, Monoisotopic m/z: 464.78223 Da (-0.15 mmu/-0.33 ppm), MH+: 928.55718 Da, RT: 22.10 min,

## Slide 8
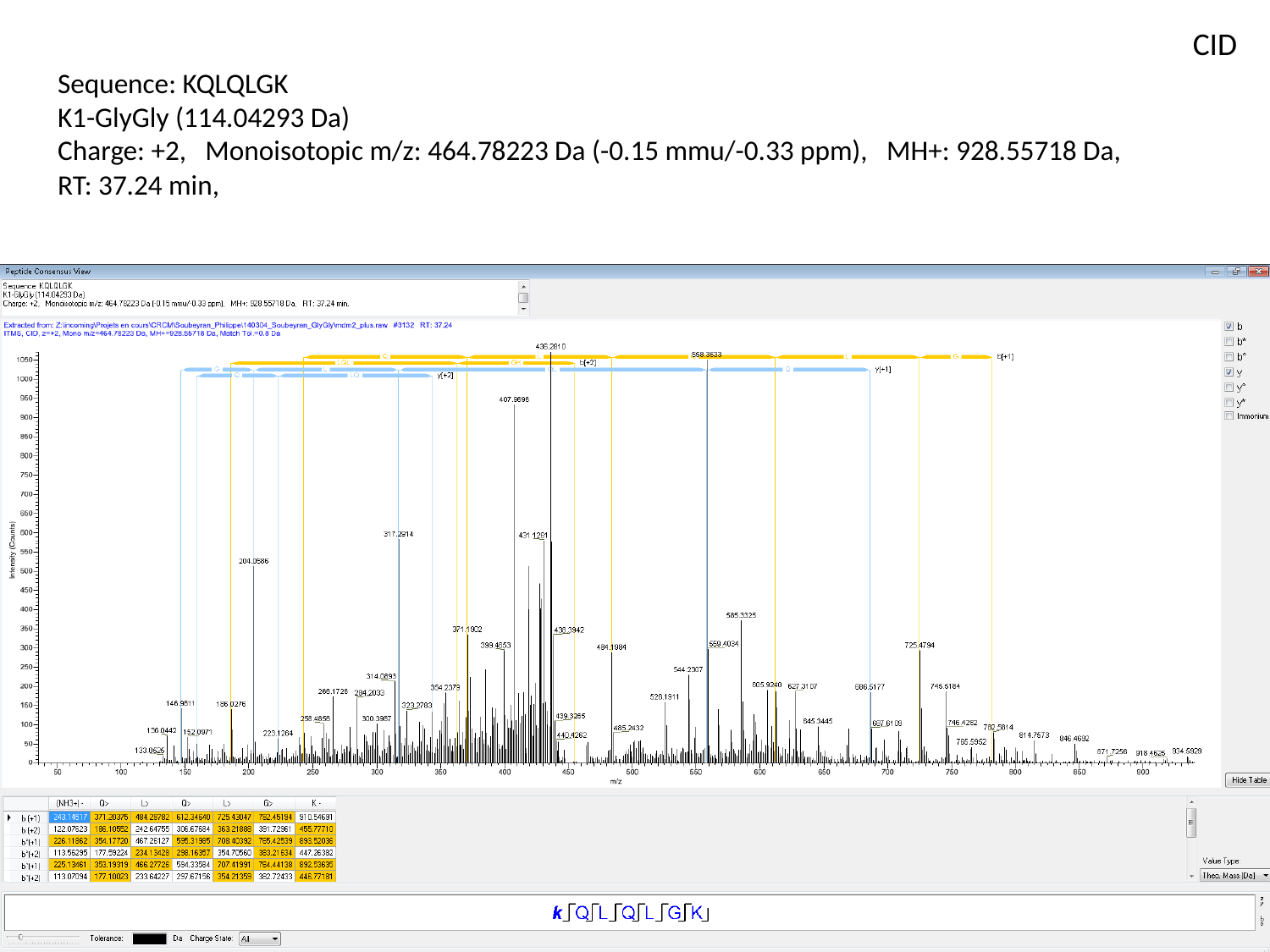

CID
Sequence: KQLQLGK
K1-GlyGly (114.04293 Da)
Charge: +2, Monoisotopic m/z: 464.78223 Da (-0.15 mmu/-0.33 ppm), MH+: 928.55718 Da, RT: 37.24 min,

## Slide 9
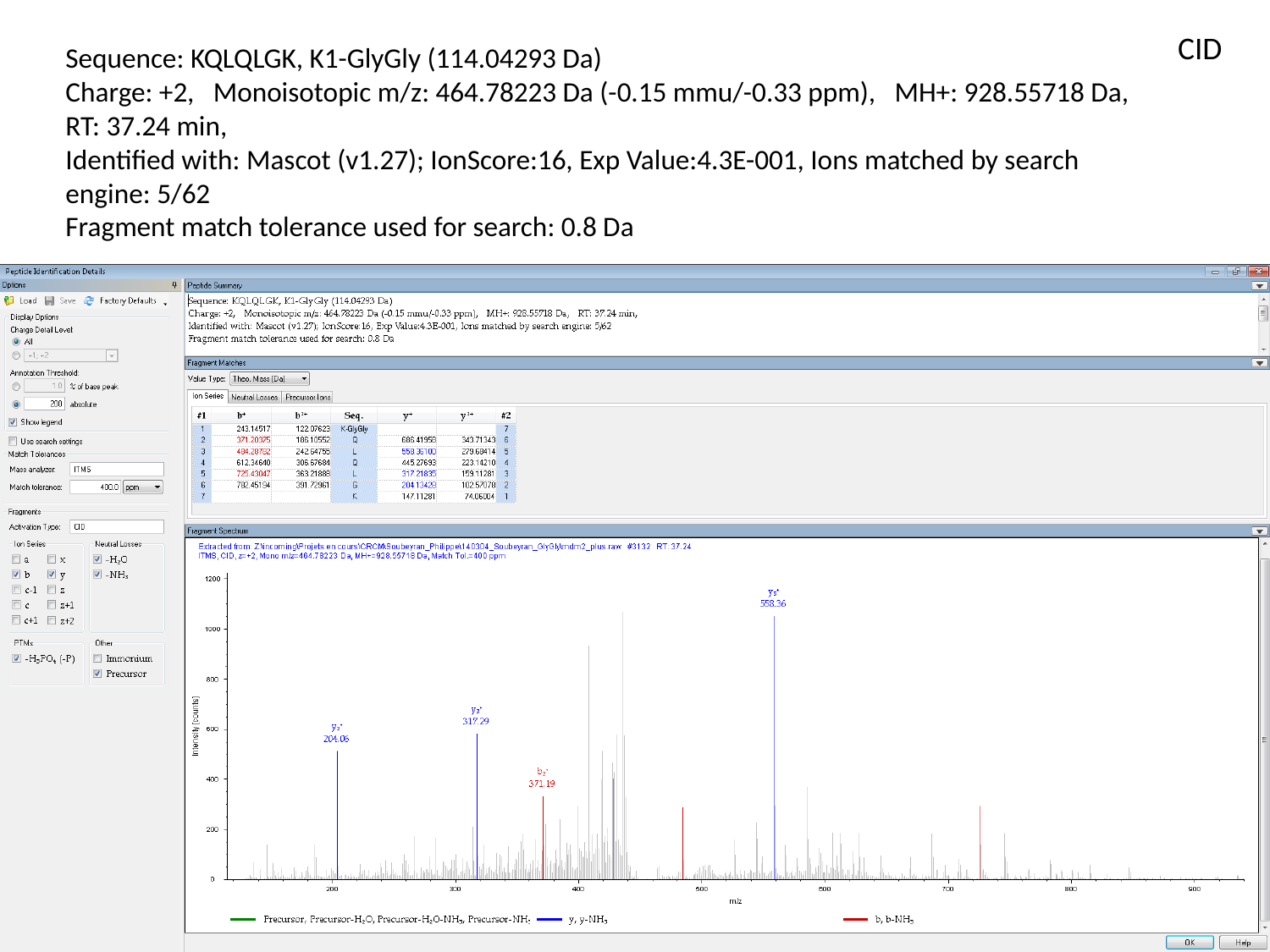

CID
Sequence: KQLQLGK, K1-GlyGly (114.04293 Da)
Charge: +2, Monoisotopic m/z: 464.78223 Da (-0.15 mmu/-0.33 ppm), MH+: 928.55718 Da, RT: 37.24 min,
Identified with: Mascot (v1.27); IonScore:16, Exp Value:4.3E-001, Ions matched by search engine: 5/62
Fragment match tolerance used for search: 0.8 Da
